# Highly sensitive salinity sensor based on Mach-Zehnder interferometer with double-C fiber

**DOI:** 10.1016/j.fmre.2021.11.023

**Published:** 2021-11-30

**Authors:** Ya-nan Zhang, Like Li, Jincheng Zhao, Yong Zhao

**Affiliations:** a College of Information Science and Engineering, Northeastern University, Shenyang 110819, China; b State Key Laboratory of Synthetical Automation for Process Industries, Shenyang 110819, China; c Hebei Key Laboratory of Micro-Nano Precision Optical Sensing and Measurement Technology, Qinhuangdao 066004, China

**Keywords:** Optical fiber sensor, Salinity sensor, Mach-Zehnder interferometer, Microfluidic channel, Double-C fiber

## Abstract

This paper proposes a highly sensitive, compact, and low-cost optical fiber salinity sensor based on the Mach-Zehnder interferometer. The sensor is constructed using a single mode fiber (SMF) - no-core fiber - double-C fiber (DCF) - NCF-SMF structure, with the DCF prepared by etching the dual side-hole fiber with HF acid. The DCF's large-size exposed microfluidic channels solve the previous microstructured optical fiber's challenging liquid filling and replacement problems. Theoretical simulations and experiments demonstrate that the sensor is suitable for high-sensitivity salinity measurement. The sensor exhibits a high salinity sensitivity of -2.26 nm/‰ in the salinity range of 10‰-50‰, as demonstrated by the experimental results. Additionally, the sensor exhibits some fascinating characteristics, including high repeatability, hysteresis, reversibility, and stability.

## Graphical abstract

**Figure:**
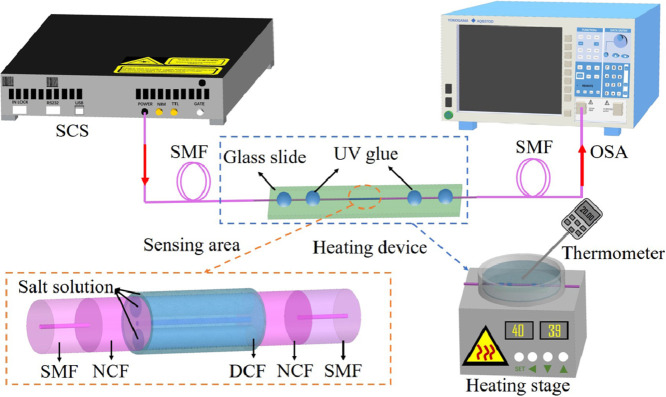

## 1 Introduction

The ocean covers approximately 75% of the earth's surface and is rich in seawater, marine food, marine energy, and marine chemical resources. The salinity of seawater is one of the most fundamental and critical physical parameters of the ocean. Theoretically and practically, measuring seawater salinity is critical for monitoring the marine environment, exploiting marine resources, predicting climate change, and ensuring military security. As a result, researchers have been interested in high-precision, high-sensitivity, and rapid detection of seawater salinity. Until now, the majority of seawater salinity detection has relied on electrochemical sensors, which measure salinity by measuring the conductivity of the seawater [1]. Although this type of sensing technology is relatively mature, it does have some drawbacks. First, the electrochemical sensor is corroded by seawater and cannot be used for long-term measurements. Second, electromagnetic signals are likely to interfere with the transmission of electrical signals, resulting in inaccurate measurement results. Finally, other non-ionic compounds in seawater are likely to affect the conductivity of seawater. Due to their attractive characteristics, such as anti-electromagnetic interference, anti-corrosion, small size, and high sensitivity, optical fiber salinity sensors have emerged as a powerful competitor since the introduction of optical fiber. They usually detect changes in seawater's refractive index (RI) to determine its salinity [2,3].

Numerous optical fiber salinity sensors have been proposed over the last several decades, including optical fiber grating salinity sensors [4,5], optical fiber surface plasmon resonance (SPR) salinity sensors [6,7], and interferometric salinity sensors [8], [9], [10], [11]. Among them, salinity sensors based on the Mach-Zehnder interferometer (MZI) are establishing themselves as a versatile and outstanding platform due to their distinct advantages of high sensitivity, low detection limit, flexibility, and compact bulk.

Several optical sensing structures based on MZI have been proposed to date, mainly classified into four categories. (1) MZI structures based on the interference of high-order and low-order modes within fibers [12], [13], [14]. To detect external RI, these structures typically rely on the mechanism whereby a change in solution RI results in a change in high-order mode effective RI. However, these structures typically have a RI sensitivity of several hundreds of nm/RIU, which falls short of the requirement for highly sensitive salinity measurement. (2) Multimode microfiber MZI structures based on the interference of HE _11_ and HE _12_ modes within the fiber [8,15]. Although these structures have a RI sensitivity of several thousand nm/RIU, they are fragile due to the microfiber waist region having a diameter of only 10 μm, which is incompatible with practical applications. (3) MZI structures based on the interference between the liquid's transmission mode and the optical fiber's transmission mode [3,[16], [17]. Typically, these structures have a RI sensitivity of up to 10,000 nm/RIU. On the other hand, these structures are typically created using a femtosecond laser (fs laser) micromachining or fiber large offset splicing. The former requires expensive instruments and extensive preparation, whereas the latter has the disadvantages of extensive preparation (offset accuracy is difficult to control), low mechanical strength, and significant spectrum loss. (4) In-fiber MZI structures based on liquid-filled MOFs [18], [19], [20], [21], [22], [23]. Due to the unique performance advantages of MOFs, these sensing structures have garnered considerable attention. First, the natural air holes along the MOF's axial direction can be used as microfluidic channels. Second, when analytes are placed inside the MOF, a large overlap area between light and analytes can be obtained, increasing the sensitivity of the detection. Finally, the inherent flexibility of MOF design enables the creation of high-performance sensing structures. However, there are two significant obstacles to the preparation of these salinity sensors. On the one hand, the microfluidic inlets must be prepared before filling the MOF with fluid because the structure is closed. Although some mature methods for preparing microfluidic inlets have been developed, such as femtosecond laser, focused ion beam, CO_2_ laser, and side polishing micromachining, these micromachining technologies are typically expensive and require complex operation procedures [24]. On the other hand, filling these MOFs with liquid typically requires the assistance of external pressurizing devices. As a result, these structures are unsuitable for detecting seawater salinity in a real environment.

As a result of the existing problems of current optical fiber MZI sensing structures, this paper proposes a novel type of MZI structure with exposed fluid channels. Single-mode fiber (SMF) - no-core fiber (NCF) - DCF-NCF-SMF is the sensing structure. At a low cost, the DCF is prepared by etching the dual side-hole fiber (DSHF) with HF acid. Compared to femtosecond lasers, focused ion beams, CO_2_ lasers, and other micromachining technologies, chemical corrosion is an effective method for ensuring an optical fiber’s smooth and uniform surface . The preparation procedure is straightforward and inexpensive, requiring only cutting, splicing, and HF acid etching. Additionally, the salt solution can be rapidly filled (within 1 second) and replaced (within 2 minutes) during the test without the use of external pressure devices. As a result, the proposed structure is capable of resolving the liquid filling and replacement issues associated with the MOF-based salinity sensor.

## 2 Sensing principle and simulation analysis

### 2.1 Sensing principle

**Fig. 1:**
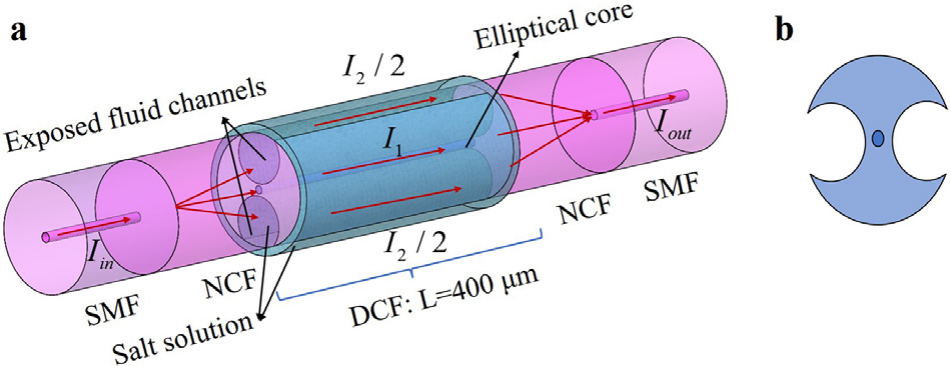
**Sensing structure and sensing principle.** (a) Schematic diagram of MZI sensing structure; (b) Cross-sectional schematic of DCF.

The sensing structure diagram of the fiber MZI is illustrated in Fig. 1. Between the two NCFs, the DCF serves as the sensing area. It is composed of an elliptical core in the center and two exposed fluid channels on either side. The two channels can operate as microcavities for transporting the seawater for measurement. The first NCF acts as the beam's separation zone, while the second NCF acts as the beam's coupling region. As illustrated in Fig. 1a, a beam of light from the lead-in SMF, with intensity $I_{in}$, will be divided into two parts when passing through the first NCF. The first part of the beam, as the reference arm with an intensity of $I_{1}$, is transmitted along the core of the DCF. The second part, as the sensing arm with an intensity of $I_{2}$, is transmitted along the seawater in the fluid channels. Finally, the reference and sensing beams will be coupled into the second NCF and then recombined in the lead-out SMF's core to generate an interference pattern.

When the salinity of the seawater in the fluid channels changes, the effective RI of the sensing arm changes, resulting in a change in the optical path difference (OPD) between the two beams. Changes in the OPD have an effect on the interference beam's output intensity $I_{out}$, which can be expressed as:

(1) $$I_{out}=I_{1}+I_{2}+2\sqrt{I_{1}I_{2}}\cos\varphi ,$$

(2) $$\varphi =\frac{2\pi L(n_{c}-n_{s})}{\lambda }+\varphi_{0},$$

where *L* represents the interference length, λ is the wavelength of the incident light, $n_{c}$ represents the effective RI of the DCF core, $n_{s}$ represents the effective RI of seawater in the fluid channels, $n_{c}-n_{s}$refers to the effective RI difference between the reference beam and the sensing beam, and $\varphi_{0}$ is the initial phase difference between the two interference beams. According to Eqs. 1,2, the output intensity of the recombined beam will reach the minimum when the incident wavelength meets the following conditions:

(3) $$\frac{2\pi L(n_{c}-n_{s})}{\lambda_{m}}+\varphi_{0}=\left(2m+1\right)\pi ,$$

where m is an integer, $\lambda_{m}$ refers to the dip wavelength of the m order interference. In general, it is assumed that $\varphi_{0}$ is 0. Therefore, according to Eq. 3, it can be deduced that:

(4) $$\lambda_{m}=\frac{2L(n_{c}-n_{s})}{\left(2m+1\right)}$$

As a result, when the length of the DCF is fixed and the effective RI of the seawater changes, the transmission spectrum's interference dip shifts. For instance, as the effective RI of seawater increases, the effective RI difference between the reference and sensing beams decreases, resulting in a blue shift in the m order interference dip. As a result, the salinity of the seawater can be determined by monitoring the movement of the interference dip.

Free spectral range (FSR) represents the distance between m order and *m*+1 order interference dip, so it can be calculated according to Eq. 4:

(5) $$FSR=\lambda_{m}-\lambda_{m+1}=\frac{4L(n_{c}-n_{s})}{\left(2m+1)(2m+3\right)}=\frac{\lambda_{m}\lambda_{m+1}}{(n_{c}-n_{s})L}\approx \frac{\lambda^{2}}{(n_{c}-n_{s})L}$$

Eq. 5 shows that the FSR is inversely proportional to the length of the DCF. The sensitivity calculation formula of the sensor can be expressed as:

(6) $$S=\frac{d\lambda_{m}}{dn_{s}}=\frac{2\pi L}{\varphi }=\frac{\lambda }{\left(n_{c}-n_{s}\right)}$$

where $n_{c}$ is approximately 1.45, $n_{s}$ ranges from 1.33-1.34. When $n_{s}$ is 1.3310 and λ is 1550 nm, we can estimate that the theoretical sensitivity of the sensor is 13025 nm/RIU according to Eq. 6. This is higher than the sensitivity of most optical fiber salinity sensors [8,^25^,26]. In addition, according to Eq. 6, we can infer that the smaller the RI difference, the higher the sensitivity.

### 2.2 Simulation analysis

**Fig. 2:**
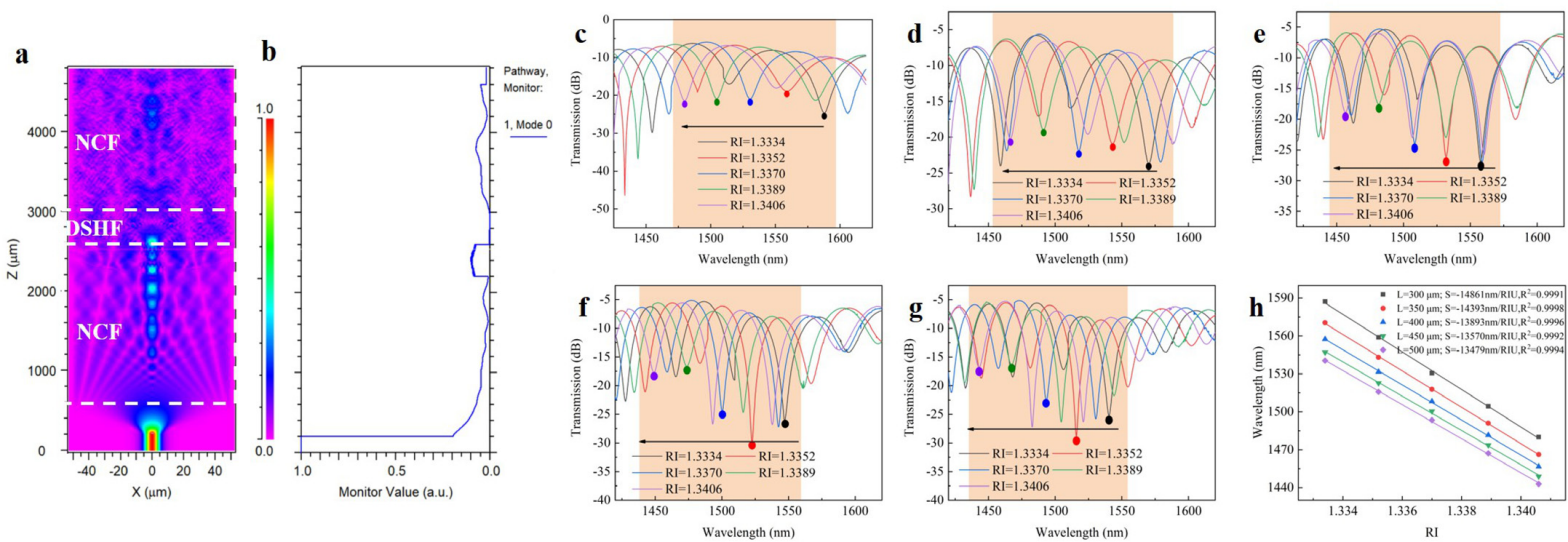
**Theoretical simulation of the sensing structure.** (a) The propagating field distribution; (b) The normalized propagating power along the beam propagation; The simulated transmission spectra under different sensing lengths ((c) 300 μm; (d) 350 μm; (e) 400 μm; (f) 450 μm; (g) 500 μm); (h) Sensitivity fitting results of different sensing area lengths obtained by simulation.

The theoretical structure was simulated using Rsoft's Beam Propagation Method (BPM) to improve sensing performance. Consider that during the actual etching of DSHF, the surrounding SMF and NCF would be corroded in the same way as DSHF. The cladding diameters of SMF and NCF were both set to 100 μm. The simulation parameters were listed in Table 1. Additionally, the DCF's holes had a diameter of 42 μm, and the distance between the center of the side holes and the center of the core was 29 μm. The interference pattern obtained by simulating the RI response characteristics of the DCF-based MZI was shown in Fig. 2a, b. They illustrated the propagating light field distribution in a medium with different RIs and the normalized light power distribution along the direction of the beam's propagation. When the sensing area was too small, the fiber structure preparation became complicated. However, when the sensing area was too large, miniaturization and integration became difficult. To investigate the optimal sensing area length, a range of 300-500 μm was chosen for theoretical simulation. Fig. 2c-h illustrated the simulation results.

**Table 1:** **Parameters of the fibers used in the simulation**.

Fiber type	Core diameter (μm)	Cladding diameter (μm)	Core RI	Cladding RI
SMF	8.2	100	1.4682	1.4628
NCF	–	100	–	1.444
DCF	9.5	100	1.45	1.445

The simulation results indicated that the performance of 300-500 μm was essentially identical when sensitivity, FSR, and fringe visibility were compared. This was also advantageous because it reduced the probe fabrication requirement for precise DCF length control. Finally, for the following experiment, the sensing length was set to 400 μm.

## 3 Preparation of the sensing probe

**Fig. 3:**
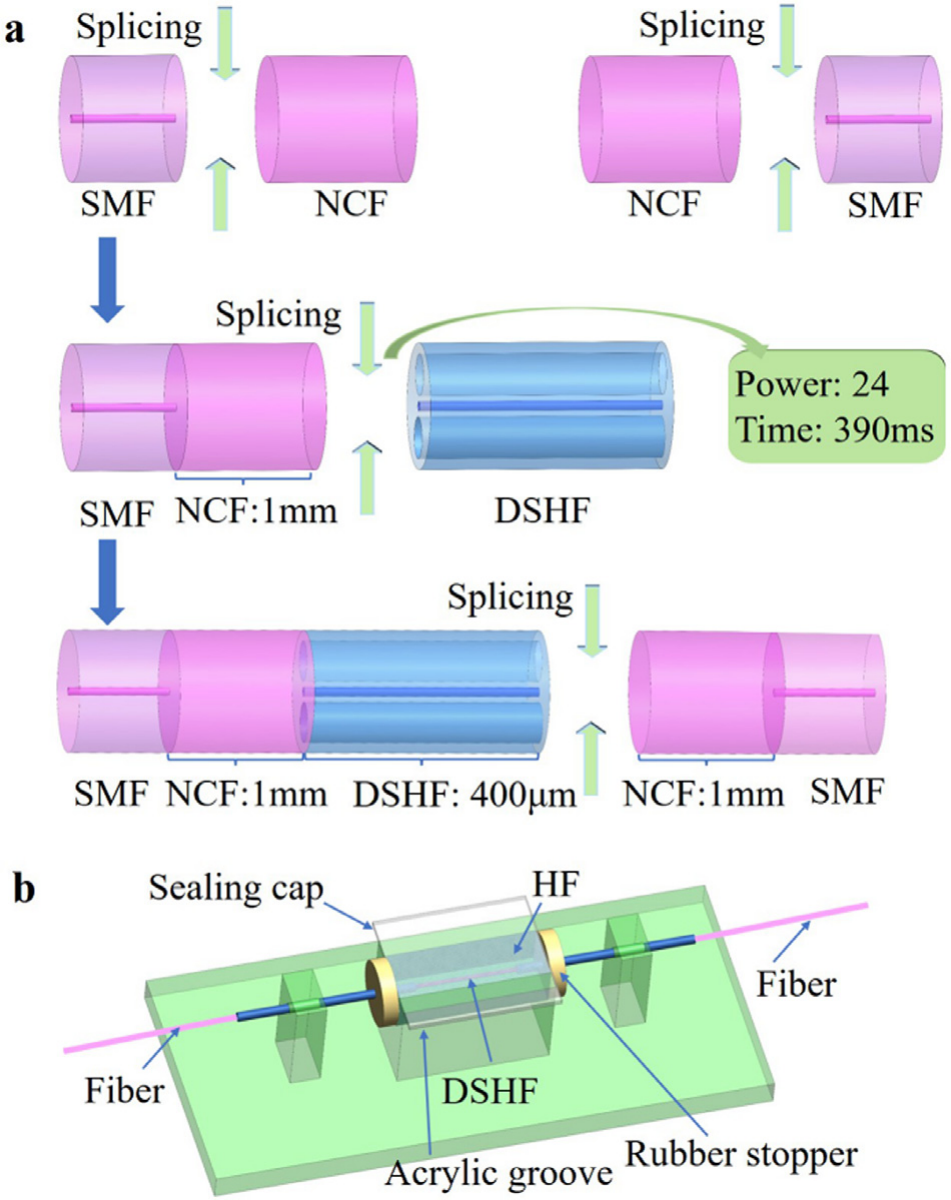
**Probe preparation procedure.** (a) Schematic of the sensing structure splicing process; (b) Schematic of the HF acid corrosion device.

The sensing probe was prepared primarily through two procedures: structure splicing and HF acid corrosion. The structure was primarily spliced using a fusion splicer (Fitel: S179C) and a cutter (Fitel: S325). Fig. 3a illustrated the splicing process schematically. First, the lead-in SMF was fused to the lead-in NCF, and then the NCF was cut to a length of 1 mm using a cutter. The same procedure was used to create a lead-out SMF-lead-out NCF splicing structure, with the lead-out NCF length remaining constant at 1 mm. Second, the NCF and DSHF were fusion spliced. After multiple attempts, DSHF could be perfectly spliced with NCF without collapsing when the discharge time was 390 ms, and the discharge strength was 24 units. The DSHF was then cut but retained its 400 μm length. Third, with the same discharge time and strength as the previous splicing, the DSHF and lead-out NCF were spliced together.

A low-cost HF acid corrosion method was used to open the side holes of DSHF in order to prepare DCF. HF acid was highly corrosive and was easily volatilized during the corrosion process. To address these issues, we designed and manufactured a simple HF acid corrosion device using an acrylic plate, as illustrated in Fig. 3b. The device included an acrylic sink, a pair of natural rubber stoppers, a fiber guide tube, a pair of holders, a sealing cover, and a base. The sealing cover prevented HF acid from volatilizing during the corrosion process, thereby preventing uneven corrosion.

The following procedure was followed for HF acid corrosion: (1) The optical fiber sensing structure was inserted into the acrylic sink via the optical fiber guide tube. (2) 40% HF acid was added to the acrylic sink, followed by the sealed cover, which allowed the HF acid to corrode the fiber for 15.5 minutes with minimal volatilization. (3) The optical fiber was gently removed from the acrylic sink using the optical fiber guide tube and then rinsed with a large amount of deionized water and ethanol to remove any remaining HF acid. (4) UV glue was used to secure the optical fiber sensing structure to the glass slide, preventing the optical fiber from being bent or broken during subsequent measurements.

**Fig. 4:**
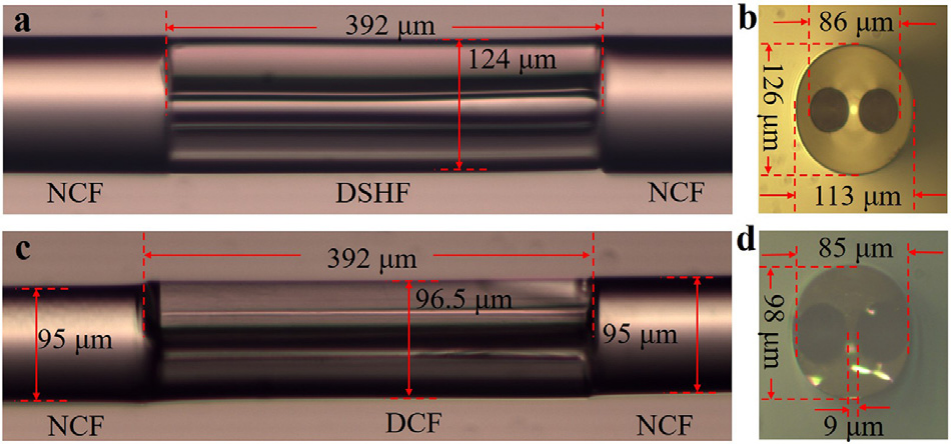
**Micrographs of the sensing structure.** (a) Micrograph of the structure after splicing; (b) Cross-sectional micrograph of the DSHF; (c) Micrograph of the sensing structure after HF acid corrosion; (d) Cross-sectional micrograph of the DCF.

Fig. 4 showed micrographs of the DSHF before and after HF acid corrosion. Due to splicing and cutting errors, the DSHF was 392 μm in length. According to the simulation results, the small length error of the DSHF would have no effect on the proposed salinity sensor's sensing properties. DSHF had elliptical cladding diameters of 126 μm and 113 μm, and elliptical core diameters of 9.5 μm and 5.8 μm. After etching, the diameters of the cladding were reduced to 98 μm and 85 μm, respectively; the diameters of the elliptical two side holes were reduced to 43 μm and 37 μm, respectively; and the distance between the center of the side holes and the center of the core was reduced to 24 μm. The two large air holes had just been opened, and there was no excess HF acid in the air holes to corrode the elliptical core at this point. As a result, the analyte could enter the air holes freely and interact strongly with the light beam in the following experiments.

## 4 Experimental results and discussion

**Fig. 5:**
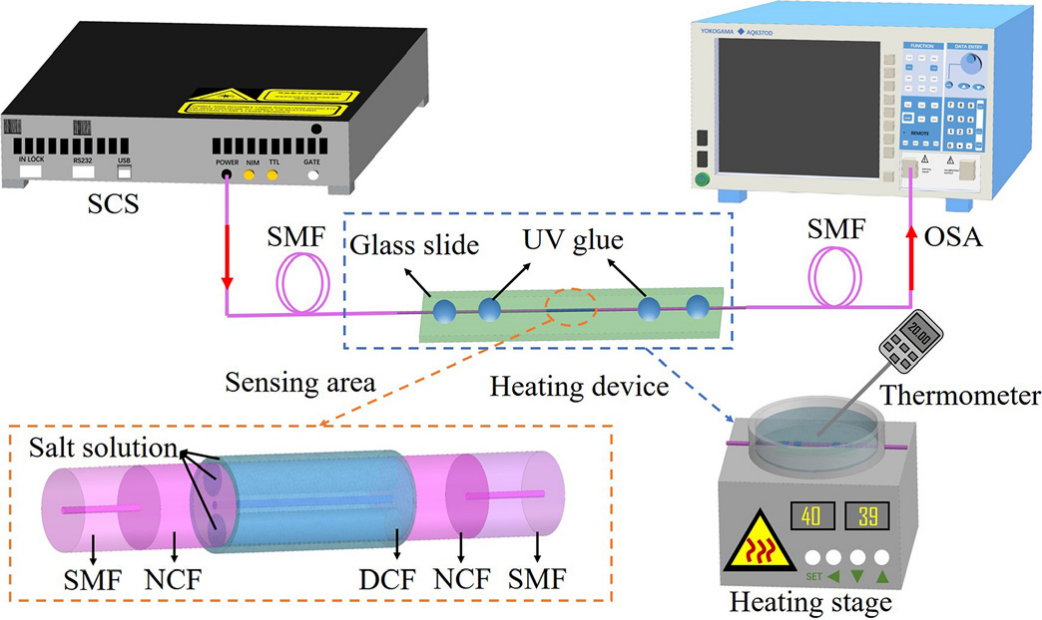
**Schematic diagram of the experimental system**.

**Fig. 6:**
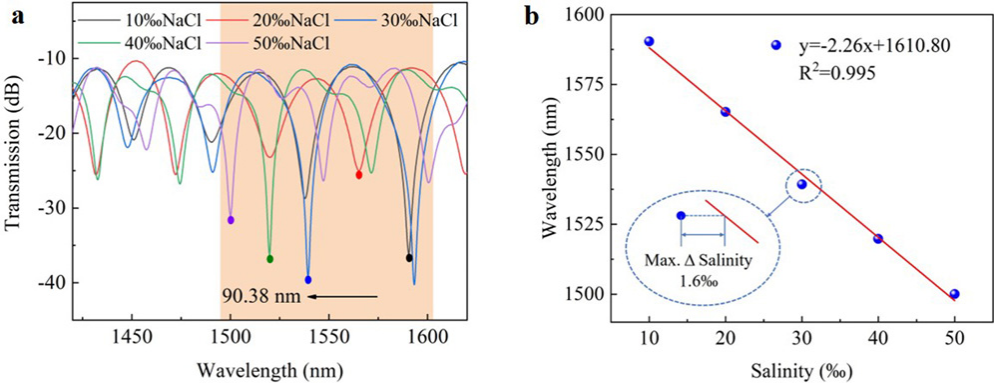
**Salinity measurement results**. (a) Transmission spectrum shifts with increasing salinity at 16 °C; (b) The function fitting of the interference wavelength with salinity.

The experimental system of DCF-based MZI was illustrated in Fig. 5, where the incident light transmitted by the supercontinuum light source (SCS, wavelength range: 400-2200 nm, light power: more than 1 W) was transmitted to the sensing structure via SMF. Then the transmission spectrum was monitored by an optical spectrum analyzer (OSA). Since the average salinity of the world's oceans is 35‰, the salinity measurement range selected in this experiment was 10‰-50‰ with an interval of 10‰. At the constant temperature of 16 °C, their corresponding RIs were 1.3334, 1.3352, 1.3370, 1.3389, and 1.3406, respectively. Numerous points near the sensing area were fixed with UV glue to prevent the optical fiber sensing area from bending and twisting during the measurement. Before the test, we used a spectrometer to observe the spectral changes that occur during the liquid filling and emptying process to estimate the structure's liquid filling and emptying time. First, after adding a drop of 0‰ salt solution in the sensing area, the spectrum changed from the air spectrum (FSR≈13 nm) to the liquid spectrum (FSR≈45 nm) within 1 s, which showed that the liquid could quickly fill the whole DCF within 1 s by the capillary effect. Then, after sucking away the salt solution around the DCF, the spectrum changed from the liquid spectrum (FSR≈45 nm) to the air spectrum (FSR≈13 nm) within 2 minutes, which showed that it only took 2 minutes for the liquid to completely empty the DCF. Following that, 10‰-50‰ salt solutions were added sequentially to the sensing area. The sensing area was rinsed 3-5 times with deionized water and ethanol before dropping the following salt solutions to improve sensing precision. The transmission spectrum was depicted in Fig. 6a. The interference spectrum exhibited a blue shift as the salt concentration increased, which was consistent with the theoretical analysis. Fig. 6b illustrated the function fitting between the interference dip and the salt solution concentration. The figure showed that the salinity measurement's sensitivity and linearity were -2.26 nm/‰ and 0.995, and the maximum error of the salinity measurement (Max. Δ Salinity) was 1.6‰ during the whole measurement. The resolution of the spectrometer used in the experiment was 0.02 nm. Therefore, the resolution of the salinity sensor was 0.009‰, which was comparable to mature electrochemical salinity sensors.

**Fig. 7:**
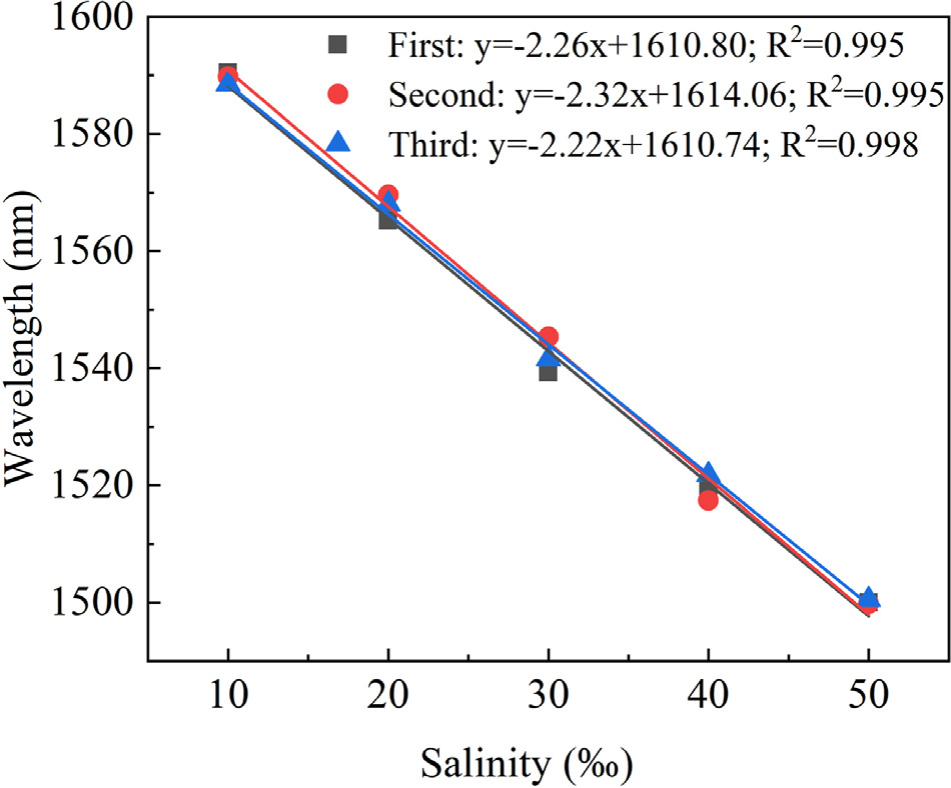
**Sensitivity fitting relationship of three repeated salinity measurements**.

Repeatability is a critical parameter of a sensor because it determines its ability to operate reliably and stably. As a result, we repeated three salinity measurement experiments on the sensor, all in ascending salinity order. Fig. 7 illustrated the results of three consecutive tests. The sensitivities of the three repeated salinity tests were -2.26 nm/‰, -2.32 nm/‰, and -2.22 nm/‰, and the linearity were 0.995, 0.995, and 0.998, respectively. Therefore, the sensor had good repeatability.

**Fig. 8:**
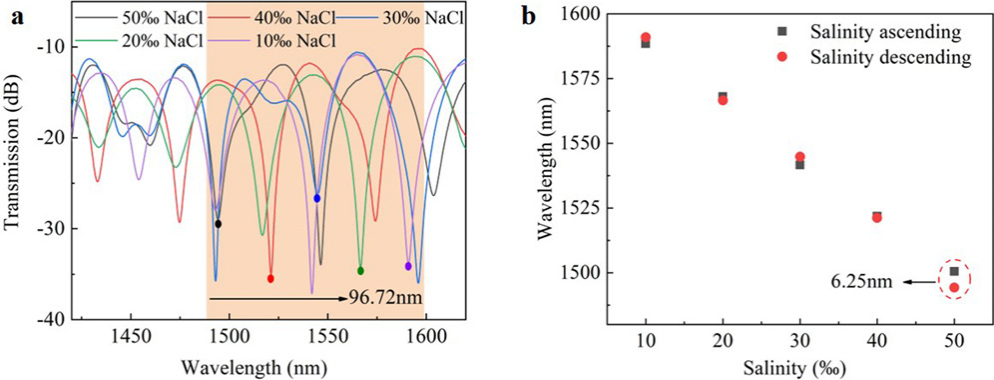
**Results of salinity's hysteretic measurements**. (a) The transmission spectrum obtained when the salinity drops from 50‰ to 10‰; (b) Experimental results of hysteresis test.

Later, the sensor's hysteresis characteristics were investigated. The transmission spectrum of the sensor was measured in the experiment in decreasing salinity sequences, as shown in Fig. 8a. Fig. 8b illustrated the interference wavelength shift experimental results in the order of increasing and decreasing salinity. When the salt concentration was 50‰, the maximum hysteresis deviation was 6.25 nm. The formula for calculating the hysteresis percentage is as follows:

(7) $$Hystersis\%=\left(\frac{{\left(\Delta Y_{H}\right)}_{\max}}{Y_{FS}}\right)\times 100\%$$

where, $\Delta Y_{H}\left(\max\right)$ is the maximum hysteresis deviation of the sensor in the process of salinity increase and decrease, and $Y_{FS}$ represents the difference of the interference wavelength caused by changing the salinity from 10‰ to 50‰. Therefore, the sensor's maximum hysteresis percentage of 6.4% could be calculated according to Eq. 7.

**Fig. 9:**
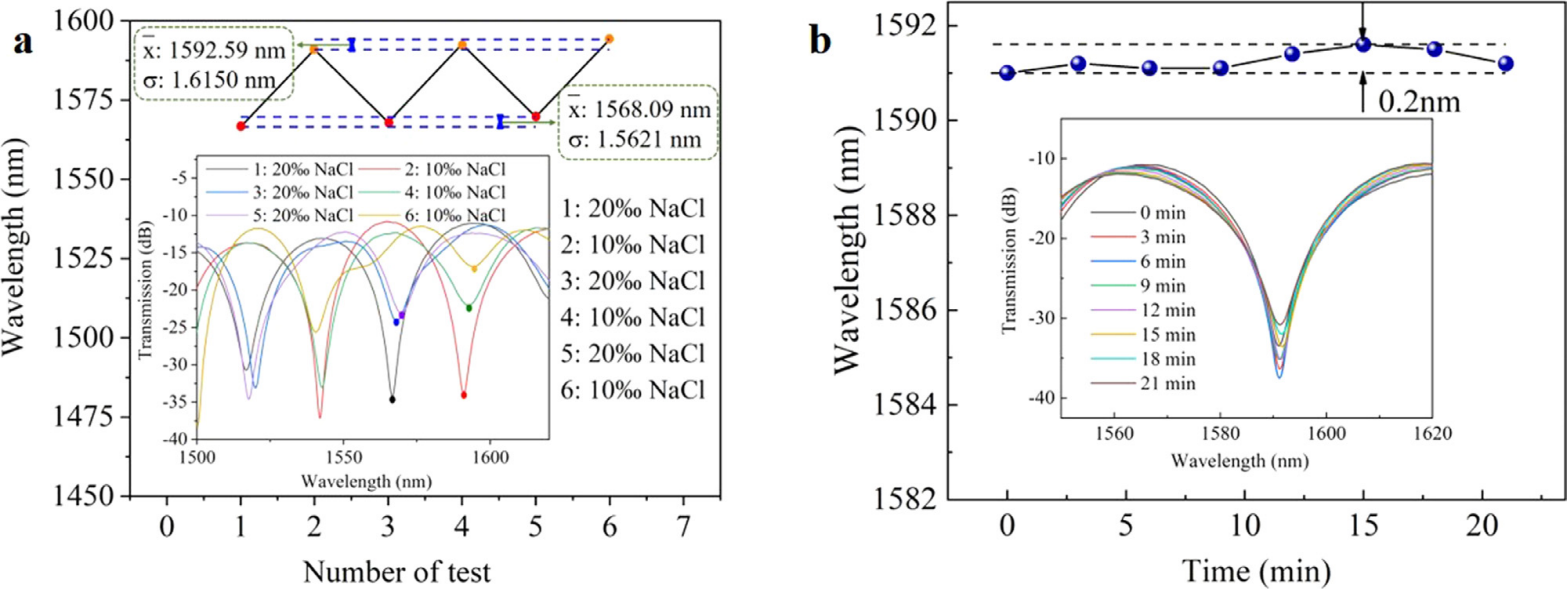
**Reversibility and stability test results of the sensing probe**. (a) Experimental results of the alternating test of 10‰ and 20‰ salt solutions; (b) The stability test of 10‰ salt solution within 21 minutes.

Additionally, the sensor's reversibility was investigated by injecting alternately 20‰ and 10‰ salt solutions into the sensing area. The results of the measurements are shown in Fig. 9a, which indicated that the sensor had an average interference wavelength ($\bar{x}$) of 1568.09 nm, a standard deviation (σ) of 1.5621 nm, and a maximum wavelength drift of 3.12 nm for the three reversibility tests of the 20‰ salt solution. As a result, the relative error could be calculated to be 3.45%. Similarly, the $\bar{x}$, σ, and maximum wavelength drift for the three reversibility measurements in a 10‰ salt solution were 1592.59 nm, 1.6150 nm, and 3.23 nm, respectively, yielding a relative error of 3.57%. The alternative measurement of 10‰ and 20‰ salt solutions demonstrated the sensor's reversibility.

Stability is a critical performance index for the sensor, as it determines the detection's accuracy. To determine the sensor's stability, we immersed the sensing area in a 10‰ salt solution. After the spectrum became stable, it was recorded every three minutes, as illustrated in Fig. 9b. Within 21 minutes, the sensor's maximum wavelength drift was 0.2 nm. Given the sensor's sensitivity of -2.26 nm/‰, the inaccuracy of the salinity measurement was approximately 0.088‰. The inaccuracy may be due to the OSA's reading error, the external environment's disturbance, or the light source's fluctuation, all of which could be ignored.

**Fig. 10:**
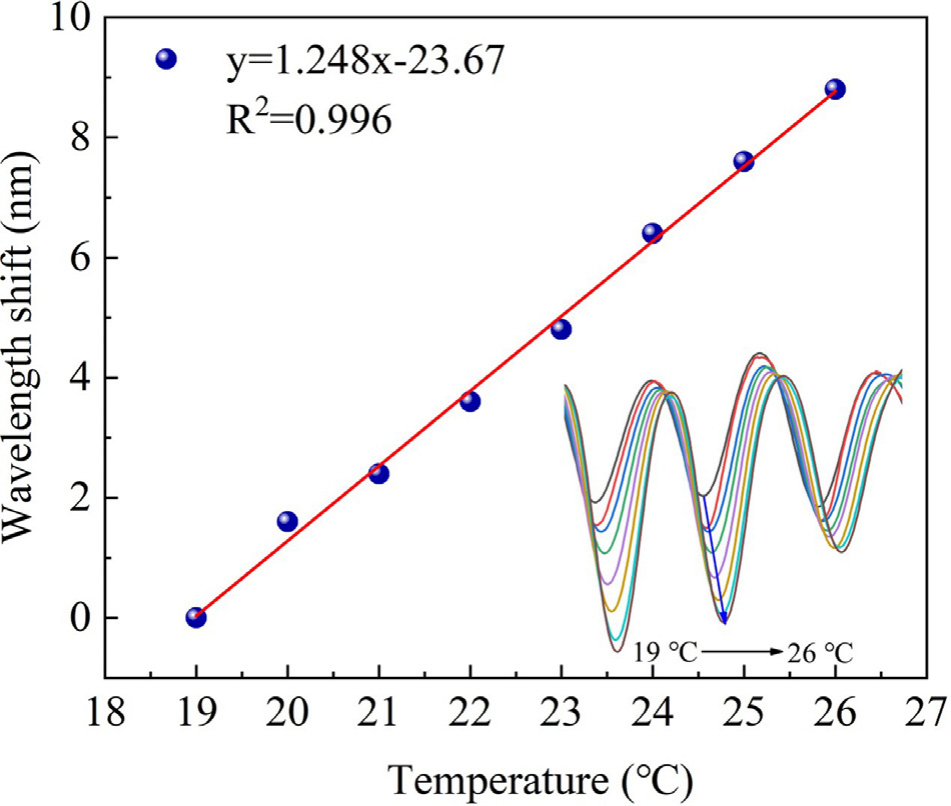
**The interference wavelength shifts as the salt solution temperature changes from 19 °C to 26 °C**.

To investigate the effect of temperature change on detection results, we heated the salt solution in a water bath. As illustrated in Fig. 5, the heating stage was used to raise the temperature of the 10‰ salt solution in the petri dish. In contrast, the thermometer was used to monitor the temperature of the salt solution in real-time. The temperature of the salt solution in the petri dish rose from 19 to 26 °C, and the spectral response of the sensor was recorded every 1 °C, as shown in Fig. 10. As the temperature increased, the interference wavelength shifted to a longer wavelength. The temperature sensitivity was 1.248 nm/°C, and linearity was 0.996. This phenomenon was explained because both the 10‰ salt solution and the DCF core exhibited a thermo-optical effect. When the temperature of the two interference arms changed, their own RI changed as well, resulting in a change in their OPD. The spectrum eventually shifted. According to the references [27,28], the thermo-optic coefficient of 10‰ salt solution and the silicon fiber core is about in the order of 10^−4^/°C and 7.8 × 10^−6^/°C, respectively. Therefore, with the increase of the salt solution temperature, the decrease of the core RI was two orders of magnitude lower than that of the salt solution and could be ignored. Thus, the spectral redshift caused by temperature increase was primarily due to the decrease in the RI of the salt solution. According to the sensor's temperature sensitivity of 1.248 nm/°C and salinity sensitivity of 2.26 nm/‰, if there is no temperature compensation in actual measurement, the salinity measurement error caused by temperature cross-sensitivity is 0.55 ‰/°C.

The proposed sensor's sensing performance was compared to published results in Table 2, demonstrating that the proposed sensor's sensitivity was greater than that of the majority of optical fiber salinity sensors. Additionally, the proposed sensor exhibited a high degree of repeatability, hysteresis, reversibility, and stability.

**Table 2:** **Performance comparison of different types of optical fiber salinity sensors**.

Sensing principle and structure	Measurement range	Sensitivity	Other performance	Reference
Long-period grating (LPG)	0‰ -190‰	- 1.0 nm/‰	–	[29]
Fiber bragg grating (FBG) coated with polyimide	0‰ - 50‰	-358 pm/‰	Good repeatability	[30]
Three-wave FPI	0‰ - 60‰	69 pm/‰	–	[31]
Microfiber MZI with a knot resonator	25‰-37‰	208.63 pm/‰	Good stability	[32]
Optical microfiber coupler	14.66‰-25.64‰	1.596 pm/‰	–	[33]
SPR sensor based on hollow core fiber (HCF)	0‰-40‰	376.9 pm/‰	Good repeatability and stability	[7]
Core mismatch and balloon shaped cascade MZI	50‰-350‰	1.683 nm‰	–	[12]
MZI based on exposed-core microstructured fiber	0‰-40‰	-2.29 nm/‰	–	[34]
MZI based on microcavity	20‰–40‰	- 2.4473 nm/‰	Good stability	[35]
MZI based on DCF	10‰-50‰	-2.26 nm/‰	Good repeatability, hysteresis, reversibility, and stability.	This paper

## 5 Conclusion

In this paper, we proposed and experimentally validated a highly sensitive salinity sensor based on DCF. Compared to other MOF-based salinity sensors, this sensor features a compact structure, high sensitivity, ease of preparation, and low cost. Furthermore, the proposed structure enables rapid liquid filling and replacement without the use of external pressure devices or microprocessors. Experimental results demonstrate that the salinity sensor has an ultra-high sensitivity of -2.26 nm/‰ in the salinity range of 10‰-50‰ with attractive repeatability, hysteresis, reversibility, and stability, which makes the sensor very suitable for high-precision salinity measurement. However, in actual measurement, the salinity sensor still faces the issue of temperature cross-sensitivity. Without temperature compensation, the salinity measurement error caused by temperature cross-sensitivity is 0.55 ‰/°C. In future research, we will concentrate on resolving the temperature cross-sensitivity problem from the design aspect of a novel MOF.
